# The Effect of POFA-Gypsum Binary Mixture Replacement on the Performance of Mechanical and Microstructural Properties Enhancements of Clays

**DOI:** 10.3390/ma15041532

**Published:** 2022-02-18

**Authors:** Abdulmajeed Alhokabi, Muzamir Hasan, Mugahed Amran, Roman Fediuk, Nikolai Ivanovich Vatin, Honin Alshaeer

**Affiliations:** 1 Department of Civil Engineering, College of Engineering, Universiti Malaysia Pahang, Lebuhraya Tun Razak, Kuantan 26300, Pahang, Malaysia; 2 Department of Civil Engineering, College of Engineering, Prince Sattam Bin Abdulaziz University, Alkharj 16273, Saudi Arabia; 3 Department of Civil Engineering, Faculty of Engineering and IT, Amran University, Amran 9677, Yemen; 4 Polytechnic Institute, Far Eastern Federal University, 690922 Vladivostok, Russia; 5 Peter the Great St. Petersburg Polytechnic University, 195251 St. Petersburg, Russia; vatin@mail.ru; 6 Faculty of Civil and Environmental, Engineering, Universiti Tun Hussein Onn Malaysia, Parit Raja 86400, Johor, Malaysia; honinalshaeer99@gmail.com

**Keywords:** soft soil, gypsum, palm oil fuel ash, treated kaolin, mechanical and shear properties

## Abstract

Soft clay is categorized as problematic due to its weak and dispersive properties which requires stabilization. In Malaysia, there is another challenge, the increment of palm oil waste productions to meet the global demand for food oil. These two concerns motivate engineers to develop novel strategies for exploiting palm oil waste in soil stabilization. Utilizing POFA as a soil stabilizing agent is an economical and sustainable option due to that POFA contains high pozzolanic characteristics which make it more suitable and reliable to treat soft soil. This study uses the replacement portion of the soil with stabilizing agents -POFA and Gypsum; aiming to achieve Malaysia green technology goals by the balance of the economic expansion and environmental privilege. However, the aim of this study is to determine the effect of POFA-gypsum binary mixture replacement on the performance of mechanical and microstructural properties en-hancements of clays. Kaolin S300 is the control sample whereas POFA and gypsum are the used binders. The mechanical properties and shear strength with the curing period were tested. Results showed that treated clay marked increment of optimum water contents and reduction of maximum dry densities, a clear 200% of enhancement of treated clay’s compressive and shear strength with curing period as well as the amount of stabilizing agent to less than 15% of POFA and 6% of POFA. It is also found that as gypsum contains a high amount of lime (CaO), the results illustrate that strength raises significantly even with less curing time due to its high reactivity compared to silica and alu-mina. Overall, the results show an enhancement of mechanical and shear strength properties of treated kaolin supported by microstructural SEM imaging.

## 1. Introduction

**Figure 1:**
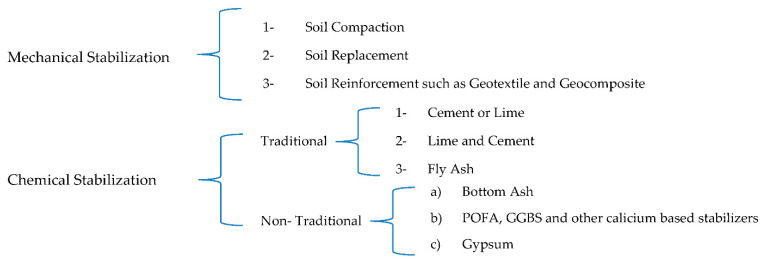
Expansive soil’s stabilizing methods.

Soft soil low capabilities to carry additional load may lead to its settlement and deformation with applying surcharge loads. Bearing capacity is one of the principles to investigate soil capability to carry and sustain loads. The presence of water in clay has an effect on its ability to shrink or swell [1]. The expansive clays swelling is caused by variations in water content, which cause severe damage to underlying buildings; civil engineering specialists are concerned about this issue [2]. Problematic soil matters can be treated by enhancing ground improvement techniques such as soil stabilization [3,4]. Soil stabilization method is an economical option to improve problematic soil properties. Generally, it is a modification technique of blending and mixing stabilizing agents with soil to enhance and improve its bearing capabilities properties in terms of mechanical compressive and shear strengths, durability, plasticity, hydraulic conductivity, and compressibility to strengthen geotechnical properties and other applications [5,6,7]. Soil stabilization agents are in a wide range and different types of various materials such as cement, lime or industrial by-product waste, when mixed with soil, they enhance soil properties because of physical or chemical effects [5]. Many materials have been utilized to stabilize soil chemically including some by-product waste such as fly and bottom ashes [8,9,10,11], cement, lime [12], eggshells [13], silica fume [14,15], and palm oil fuel ash [7]. Various research has used wastes materials to enhance road surface strength as well as different geotechnical applications [6,16]. There are also various types of methods and technques used for soil stabilization, for instance, chemical and mechanical stabilizations [17,18]. Figure 1 shows some of the methods.

Due to daily life activities, huge volumes of by-product materials are created all over the world [19,20,21,22,23,24]. They have a detrimental effect due to the potential disposal cost and contamination to land as well as groundwater which is triggered by heavy metals, which are considered to be a part of the POFA waste chemical composition, which leads to deterioration on sustainability and the environment [25,26,27]. The practice of utilizing industrial by-product waste became well-known worldwide [28]. Palm oil is considered as the Malaysian fourth gross national income (GNI) and the first most important and sustainable vegetable production in the world [29,30]. It is also reported that the largest production of POFA is recorded in east Asian countries (Indonesia, Malaysia, and Thailand) as well as West African (Ghana, Nigeria, and the Benin Republic) [31]. Malaysia and Indonesia produce the main palm oil demand, manufacturing 86% of the globally demand stated by many researchers globally [7,32,33,34]. In 2013, Malaysia had planted about 5.23 million hectares of oil palm trees. Sabah was occupied with the largest oil palm area of 1.48 million hectares (about one-third of the total area), Sarawak also planted with 1.16 million hectares (which is about 23.2% of its area) [30]. Malaysia is encouraging the initiatives of zero waste as a part of the National Biomass Strategy 2020 (NBS2020), which is focused on palm oil biomass [29]. Malaysia ranked the productions of the oil palm biomass as the largest waste in the country, where a huge amount is useless [35]. In 2012, Malaysia produced about 143 million tonnes of solid and liquid POFA waste [29]. POFA is a usless waste material produced from the palm oil mills; it is mentioned that Malaysia produces about 5 million tonnes of POFA waste [13,36]. The landfill and dumping action of POFA to open areas triggers the issues of environmental contamination [37]. POFA waste negative impact is not limited to the environmental and sustainability effect of potential land and air pollution, as well as groundwater contamination, which is caused by the chemical composition of heavy metals, but it can include the cost of disposal and transportation [25]. As a result of the rapid increment in the palm oil industry, the ash produced has taken a significant impact on the environment and using it properly is a national goal [38,39]. Engineers address these issues with some urgency to minimize them both environmentally and financially [22,24,26,40]. Engineers have adopted new methods to utilize the waste to achieve the aim of sustainable development, which requirew Reduce, Recycle and Reuse (3Rs) [25,41,42]. On the other hand, POFA contains high pozzolanic characteristics which make it more suitable and reliable in treating soft soil [43,44]. POFA has a high potential to be used as a technique of soil stabilization due to its high siliceous content, which encourages the pozzolanic reactions [38], the reaction of which produces calcium aluminate hydrates and stable calcium silicate hydrates. POFA is preferred as a stabilizing agent of soft soil more than traditional calcium-based binders because of its suitable properties, its low environmental issues, its low processing costs, and its sustainable solutions for waste [45]. Utilizing this method of POFA replacement can contribute to managing the waste and reusing it in a better, more sustainable way, as well as to improve the soft clays engineering and strength properties. The newly adopted methods combined the enhancement of soil properties by utilizing waste and reducing landfill and heavy metals contamination. This method of utilizing biomass of by-product waste is used to tackle issues of the daily massive production of solid waste and problematic soil.

Gypsum by-product is considered scheduled waste in Malaysia and in many other countries [46]. Gypsum is a mined substance and has many products that are utilized in the construction industry and agriculture [47,48,49]. Gypsum properties are better than organic binders because they have no impact on air pollution. Gypsum is cheaper than portland cement, is fire-resistant, and is reluctant to the biological and chemical factor deterioration [50]. Gypsum is counted as a by-product of various industrial processes. Both coagulation and cementation of the soil are probable to be achieved by gypsum and lime or gypsum and cement addition, which leads to a significant improvement in soil structure [47]. Gypsum can be used as a soil modification method to improve crop yields, soil characteristics and soil structure [1].

## 2. Theoretical Background

Gypsum is one of the materials employed for chemical improvement as an alternative technique by many researchers [51,52]. Gypsum is also the main source of sulfates and reacts to form ettringite in alumina-rich soil, which is the resulting compound shown in Equation (1). It also has a high calcium ion concentration which accelerates the pozzolanic reaction [53]. Gypsum acts as a cementing agent within soil particles, leading to an obvious increment in soil cohesion properties [54].

Ca(OH)_2_ + NaSO_4_ + 2H_2_O → 2NaOH + CaSO_4_·2H_2_O (1)

Gypsum decreases soil loss and absorbs more water, identified as the ionic strength effect [55]. The high gypsum amount should be considered in soil, where the high amount may increase the potentiality of internal sulfate attack [56]. The usage of fly ash and gypsum in peat stabilization shows that the unconfined compressive strength (UCS) was enhanced with the curing period increase [57]. The chemical characteristics of gypsum are containing a high CaO amount, which is considered as one of the key factors in improving bonding properties between the particles of clay [58]. The UCS increases with the gypsum addition to the clay soil, but the UCS is decreased after adding more than 6% of gypsum [57].

When a stabilizing agent is blended with clayey soil, the change process takes four sequential phases. The first two phases are defined as modification stages and the other two are considered as stabilization stages, cation exchange is the first stage which is followed by agglomeration and flocculation, which is caused by water reduction, pozzolanic reaction is the third sequential stage, and lastly the self-healing process [59]. Two main factors affect on soil stabilization process, which is mixed design (stabilizer proportions) and curing time (considered as one of the most significant factors that influence the extent of soil stabilization) [60,61]. Researchers concluded that some of the by-product waste materials which possess a high lime content and a high amount of silica and alumina may assist to improve soil characteristics. POFA-soil modification/stabilization is dependent on the physical properties of POFA, as well as gypsum and the original soil, and the interactions between the minerals of kaolin and the POFA-gypsum mixture for fly ash, which also interacts differently, even from the same source [62].

The key item of Portland cement hydration is calcium silicate hydrate gel (C-S-H). It is mentioned that Ca, Si, Fe, and Al are the basic chemical elements of cement and their presence ratio is critical in producing a greater strength enhancement during the hydration process when mixed with water [63,64]. When the silicate phases of C3S and C2S interact with water, two main products will be formed—crystalline calcium hydroxide and the highly disordered amorphous C-S-H—which constitute over 60% of the hydration process [65]. The hydration process result shows that SiO_2_ and CaO are the most important chemical compositions in increasing the mechanical strength bonding [16], where C_3_S (3CaO∙SiO_2_) reacts quickly with water and generates a relatively high heat to form C-S-H (calcium silicate hydrate) as an early strength of cement, C2S (2CaO∙SiO_2_) for ultimate age strength enhancement. C_3_A (3CaO∙Al_2_O_3_) and C4AF (4CaO∙Al_2_O_3_∙Fe_2_O_3_) also participate in the early set of hydration and ultimate age strength [32,66]. If the sum of SiO_2_ + Al_2_O_3_ + Fe_2_O_3_ is more than 50% but less than 70%, it is considered a class C pozzolan according to ASTM C618 [44]. During the hydration process, two main products of CSH (3CaO∙2SiO_2_∙3H_2_O, the most important product) and hydrated lime are formed. The generation of hydrated lime (Ca(OH)_2_) during the hydration process is an important key for increasing the pH; thus, when pH ≥ 12.4, it leads to hydrated lime ionization of the additives (binders) as shown in Equation (2)

Ca(OH)_2_ → Ca^2+^ + 2(OH)^−^ (2)

In the soil system, clay is the main soil component as it controls chemical reactivity due to its fineness and large surface area [46]. Moreover, if pH ≥ 10.5, the minerals of kaolin clay are dissolute, as in Equations (3) and (4).

Al_2_Si_4_O_10_(OH)_2_·nH_2_O + 2(OH)^−^ + 10H_2_O → 2{2Al(OH)^4 −^ + 4H_4_SiO_4_} + nH_2_O (3)

2H_4_SiO_4_ → 2H_3_SiO_4_^−^ + 2H^+^ → 2H_2_SiO_4_^2−^ + 2H^+^ (4)

The products of alumina and silica will interact with ions of calcium from POFA and gypsum to produce two types of cementing agents, CSH {3H_2_O·3CaO·2SiO_2_} and CAH {(12H_2_O·3CaO·Al_2_O_3_·Ca(OH)_2_)}. It is reported that increasing pozzolan fineness increases the strength activity index [67]. The increase in strength depends on the SiO_2_, Al_2_O_3_, CaO, and pozzolanic effects, and the chemical action of NaOH in cement where the POFA shows a slow enhancement of strength in the presence of NaOH as a result of the poor chemical composition of CaO and Almunia. Gypsum has been used as a pozzolanic activator with fly ash as an external source of Ca as PC, or gypsum with a higher amount of hydrated lime (Ca(OH)_2_) and is expected to result in the production of ettringite and crystalline [68]. The production of silica and alumina from a soil chemical reaction will react to form the cementing agents, where the pozzolanic reaction of POFA occurs based on Equation (5) according to Ouhadi et al. [36,69] and Equation (6) according to Ouhadi et al. [69]

Ca(OH)_2_ + SiO_2_ (POFA) + H_2_O → CSH {3CaO·2SiO_2_·3H_2_O} (5)

Ca^2+^ + 2OH^−^ + Alumina ions → CAH{(3CaO·Al_2_O_3_·Ca(OH)_2_)·12H_2_O} (6)

This research concentrates on studying the effectiveness of the replacement of POFA and gypsum in the stabilization of soft clays in terms of the compaction properties of maximum dry density and optimum moisture content, as well as compressive and shear strength properties. kaolin S300 was used in the treatment because of its high settlement, low strength in water presence and because as it is easily dispersed in water [70,71]. Moreover, it has poor and expansive geotechnical properties, a low workability and a high plasticity [72,73]. POFA is potential waste to be used as a stabilizing agent due to chemical compositions such as Aluminia and Silica; it is also used as a technique in waste management [74,75]. Gypsum was added as a stabilizer and a pozzolanic activator [68]. To treat kaolin soft clay by the addition of a POFA-gypsum mixture to modify its geotechnical properties, the improvement was evaluated and monitored based on POFA various percentages. It showed better results in terms of compaction properties as well as shear and compressive strength.

## 3. Materials and Design

The used kaolin is a powder grade S300 in this study. Kaolin is imported from Selangor, Kaolin Malaysia Sdn. Bhd. In this experiment, kaolin is hydrous silicate-alumina and it has the general chemical of formula Al_2_(Si_2_O_5_)(OH)_4_. Malaysia has huge deposits of kaolin, with appoximately 112 million tonnes [76]. Kaolin is a tropical intricate soil and because of the seasonal water inconsistency, it is exposed to a volumetric change [72]. It is chosen to be stabilized due to its poor geotechnical properties, its expansive condition, high plasticity, low shear strength, and low workability [72,73].

POFA waste is a by-product ash generated from empty fruit bunches and shell combustion in the boilers of palm oil mills. Shells and empty fruit are heated at the estimated temperature of 800–1000 °C to produce steam. During the milling operation, the generated steam is exploited as an energy source and is used in turbines to provide electricity [77]. POFA is shown in Figure 1, which is a pozzolanic waste material, collected from Lepar Hilir Palm Oil Mill, Gambang, Kuantan, Pahang, Malaysia. The huge amorphous silica amount as chemical composition in POFA potentially initiates and contributes to the pozzolanic reactions during the hydration process, which produce and generate cementations compounds known as calcium silicate hydrates (CSH) and calcium aluminate hydrates (CAH); these compounds are in charge of enhancing the engineering characteristics of soil, which develop over time in reactions called the pozzolanic reactions [25].

**Figure 2:**
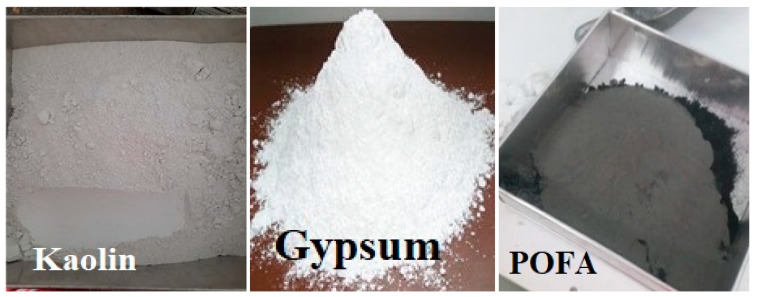
Used POFA, kaolin, gypsum in experimental laboratory work.

Gypsum is a white material that contains hydrated calcium sulphate. It was imported from Kiong Gay, Johor, Malaysia. The chemical formula of gypsum is calcium sulphate dihydrate (CaSO_4_·2(H_2_O)). It is a naturally produced material that constitutes water and calcium sulphate. It is also produced as a byproduct of various industrial operations and processes. It is also sometimes referred to as hydrous calcium sulphate. POFA, kaolin and gypsum samples are shown in Figure 2. The colour of any material is dependant on its minerals; therefore, gypsum is a white powder, POFA is black in colour ash and the finer the POFA, the more greyish it is in appearance. Kaolin has a creamy to white colour. As a texture classification, kaolin is soft, very fine, and smooth, POFA is gritty sandy in texture, whereas in smaller particles than 0.425 mm, it seemed to be wet and smooth, whereas gypsum had a gritty silty sand texture. Table 1 shows the utilized material chemical compound percentages.

### Test Preparation and Procedures

**Figure 3:**
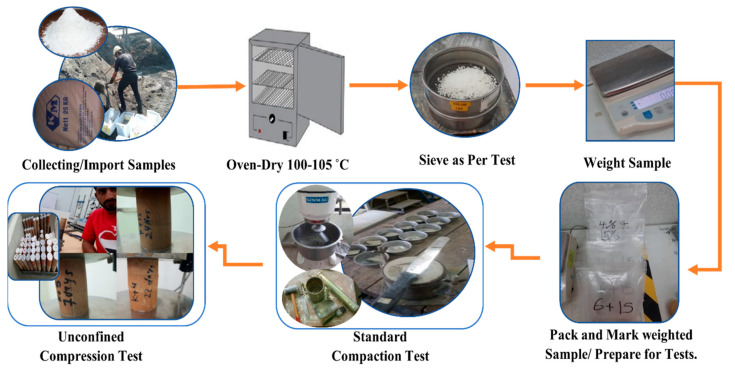
Experimental laboratory workflow.

This test was mainly planned to determine and investigate the variation of compaction characteristics of the control clay sample and the treated clay with gypsum and POFA. The soft soil used in this study was kaolin powder; it was substituted with different percentages of POFA and gypsum. The flow of experimental work was in accordance with standard laboratory procedures and results analysis methods, according to the American Society for Testing and Materials (ASTM) and British standard (BS). The standard compaction test was executed according to BS 1377:1975 to identify and determine the main compaction parameters (maximum dry unit weight and optimum moisture content) [78], where the optimum water content was determined from SPT to be used for an unconfined compression test (UCT). UCT was carried out according to ASTM D 2166 and BS 1377-7:1990 [79,80]. Figure 3 shows the flow of laboratory work.

The amount of gypsum and POFA required is controlled by kaolin S300 dry mass. All materials are oven-dried for 24 h at 105 °C. Following that, the materials were sieved in line with the British standard by using a particular sieve size per test (BS). Before testing, the produced mixture should be well stirred with a soil mixer until it seems homogeneous. The gypsum percentages (4 and 6%) were chosen based on a study of existing work, whereas the POFA percentages (5, 10, and 15%) were chosen at random to test POFA content in varied proportions with gypsum to treat and stabilize kaolin and investigate the improvement on geotechnical mechanical characteristics.

This test procedure was conducted as presented in Figure 3, the procedures followed with BS1377:1975 and ASTM D 698 [81]. The standard proctor test is executed to determine the relationships between various water content and compacted dry densities of kaolin clay. The imported and collected materials were sieved and were finer than the 4.75 mm sieve after oven-drying for 24 h at 105 °C. The mixture of materials was initiated and then water was poured and mixed with the homogeneous mixture. Water was added up in a sequent manner of 5% until the moulded mixture mass showed a loss of weight; water usually plays the softening role in mixture particles. With softening and compaction, the mixture particles are brought nearer to each other as they are forced to move into a dense situation. The sample is usually compacted into three layers with equivalent thickness to a metallic cylinder of about 105 mm inner diameter and a volume of almost 986 cm^3^. The compaction was made by a 2.5 kg metal rammer and had a diameter circular face of 50 mm, drops of 25 blows and a height of 300 mm into the mould.

The unconfined compression test is well known and is systematically used in many experiments presented in the literature review to prove and establish the effectiveness in soil stabilization [82]. UCT is a special triaxial test; the confining pressure is negligible to be considered as zero value. It is the most popular method of soil shear testing to evaluate shear and compressive strengths of the soil and its suitability to evaluate various civil engineering projects because of its simplicity, quickness, promptness, and cheaper price for measuring soil strengths. It is an approach that is utilized to determine the UCS and stress–strain characteristics of the fine-grained soils. Typically, the test is suitable for measuring the compression-loading on cohesive samples.

UCT was performed to study and evaluate the performance of different stabilizing agents on the increment of strength after stabilization with time. The procedure of conducting an unconfined compression test can be found in ASTM D 2166 and BS 1377-7:1990 [79,80]. The specimens tested for these experiments were prepared by compacting the mixture of the used stabilizers and soil with the optimum water content of the kaolin with different precentages of gypsum and POFA, as presented in Table 2. The cylindrical specimens were tested in compression, as there was no lateral support by using an unconfined compression test machine, as in Figure 3.

The UCT Metallic loading frame has two plates. The upper plate is fixed and united to the measuring device of the load, connected to an electronic load cell or calibrated proving ring. After placing the compacted sample between both plates, the moveable bottom plate is progressively raised. The resistance provided by the fixed top plate initiates and applies an axial force on the compacted specimen. The load is measured by an electronic load cellor and a calibrated proving ring. Vertical deformations were determined by a dial gauge. The dial gauge is linked to the upper plate and measures the relative motion between the fixed and bottom plates. As the bottom moveable plate was risen, an axial load was produced on the compacted specimen at a constant strain rate. The specimen was loaded until it failed, as it exceeded its UCS. The sample slowly sheared with a gradual rise in load. Readings were taken every 20 s and began directly when the force was applied to the sample. At any stage, the samples were considered to have failed when the axial stress at failure was the unconfined compressive strength. The load–deformation curves were plotted in axial strain versus axial stress. The measured data determined the strength of the kaolin and treated the kaolin specimen and stress–strain data. UCS is the maximum load per unit area.

## 4. Results and Discussion

### 4.1. Optimum Water Content and Maximum Dry Density

**Figure 4:**
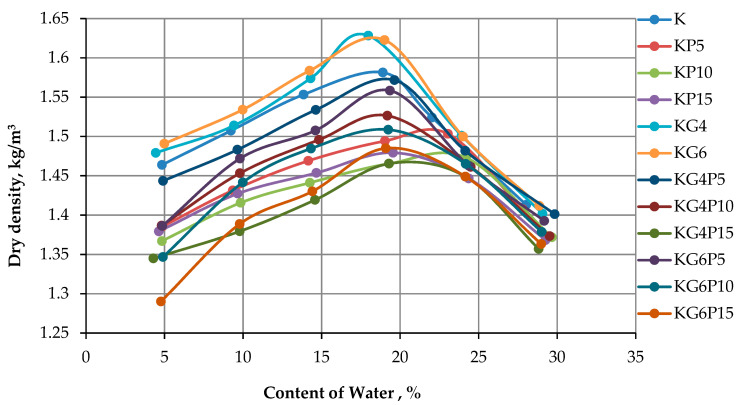
Variation of MDD and OMC of solo kaolin and stabilized kaolin with POFA and gypsum.

The efficient compaction is defined by four parameters: water content, dry density, soil type and compaction type (light or heavy). The compaction curve was created and plotted against the dry densities and water content added to the mixture sample for each cycle, as shown in Figure 3. The optimum value of the curves is the significant point; it highlights the OMCs and the MMDs as reported in the published work by Alhokabi et al. (2021) [7] From Figure 4, the OMC was raised with the substitution of the POFA content. Therefore, for treated kaolin, the more POFA substituted, the higher the OMC achieved; on the other side, the MDD is decreased with the increasing POFA content. It can be explained that POFA has a low specific gravity (2.25) counterweighted with kaolin clay and gypsum [83]. The results showed an increase of OMC of the treated soft clay with POFA; the results are in line with previous reported works [83,84,85,86] and the referred increment can be explicated as the calcium ions, which are released from POFA and were crowded out during the ionic dissociation of hydrolyzed calcium oxide during the pozzolanic reaction between POFA calcium ions and kaolin SiO_2_ where both identified as factors of the chemical stabilization of soft clays. The high lime amount in gypsum could be the reason behind the water absorption, soaring in treated kaolin where it has been stated that lime is technically known to reduce the plasticity index and soil MDD and increase its OMC [6].

It also exhibited a drop of MDD and an increment of OMC of the stabilized samples compared to the control sample can be predicted as a result of higher water absorption, which occupies the pore space of the compacted sample and leads to the buoyancy of the particles [84], the more amount of fibre and the specific gravity of the substituted POFA also effected on the drop of maximum dry density [83,87]. The reduction of MDD might be to the compaction resistance initiated and triggered by the flocculation of mixture particles during soil stabilization [60]. Previous studies on POFA utilized as a stabilizing agent with lime found that the density of mixture was affected by lime content, where the lime tends to decrease the maximum dry density of stabilized sample as a result of water suction and the absorption of treated soil [51,58,84,87,88,89].

### 4.2. Stress–Strain Curve of Unconfined Compression Test

The strength of the mixture depends on different factors, for instance, admixture content, soil compositions, admixture type, water content during stabilization, curing period, and mixing process. The results in this part show the curves of the unconfined compressive strength of kaolin and treated kaolin specimens mixed with various contents of POFA and gypsum in different curing periods of 0 days (testing conducted directly after compacting specimen), 1 day (24 h), 7 days, and 28 days. Overall, all figures illustrate that the UCS in all treated samples increases with the increasing binders’ content and curing period.

Overall, the results show satisfactory UCS and shear strength enhancement with the addition of gypsum and POFA to a certain percentage of POFA. These research results are in line with previous research results conducted by Pourakbar [38], although he has used a mixture of cement with POFA. It is found that the increase in the Ca^2+^ ion concentricity in soil due to gypsum substitution accelerates the soil–lime reaction and enhances the soil cementation, which might also happen to POFA and gypsum to enhance soil strength [90]. The increment of the strength with the mixture of gypsum and POFA is also predicted due to soil susceptibility to the amount of water variation reduction. This is followed by agglomeration and flocculation of soil particles [60].

**Figure 5:**
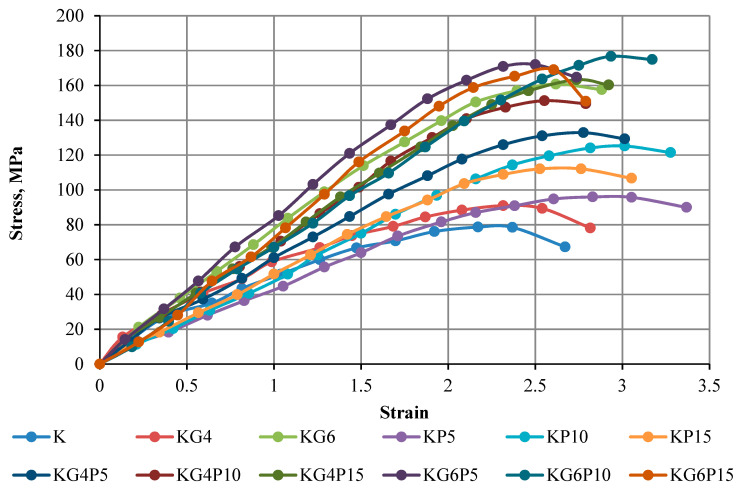
Stress–strain curve of kaolin and treated kaolin with gypsum and POFA with no curing—on the 1st day.

**Figure 6:**
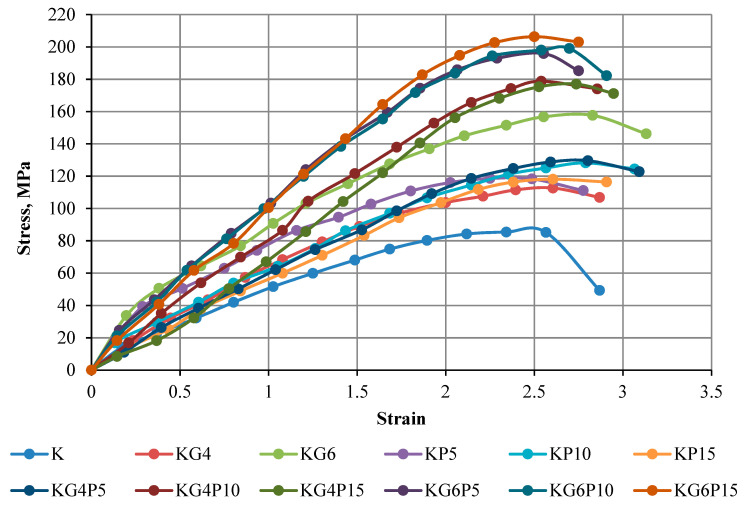
Stress–strain curve of kaolin and treated kaolin with gypsum and POFA tested after 1 day of curing.

**Figure 7:**
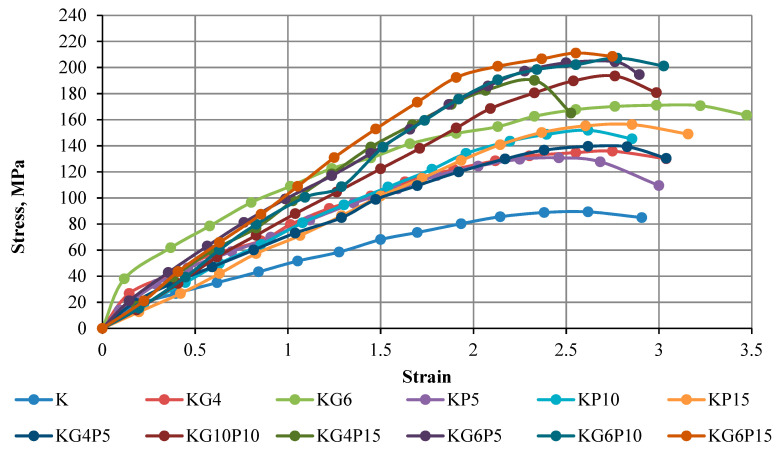
Stress–strain curve of kaolin and treated kaolin with gypsum and POFA tested after 7 days of curing.

**Figure 8:**
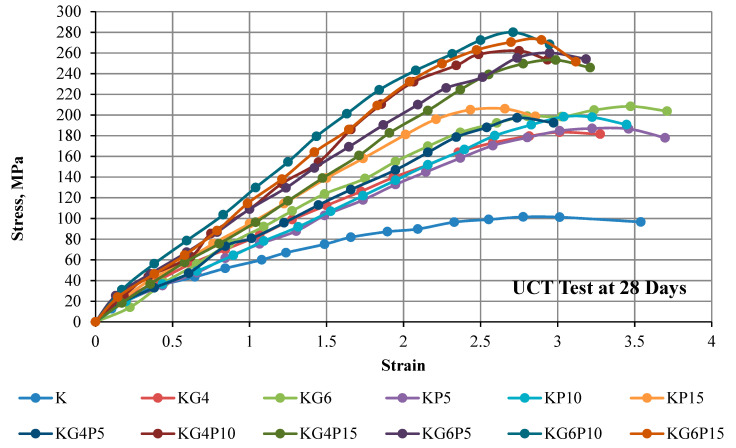
Stress–strain curve of kaolin and treated kaolin with gypsum and POFA tested after 28 days of curing.

The findings in Figure 5, Figure 6, Figure 7 and Figure 8 of this experimental work illustrate that a combination of gypsum and POFA yields a higher compressive strength than POFA alone or gypsum alone in different days of curing. In brief, it can be noted that utilizing the combination of POFA and gypsum in soft soil stabilization undoubtedly facilitates a lowering of the impacts of industrial by-product waste of POFA on the environment for sustainable development in line with reducing construction costs on the basis that gypsum has no negative impact on the environment.

### 4.3. Shear Strength with Percentages of the Stabilizing Agents

**Figure 9:**
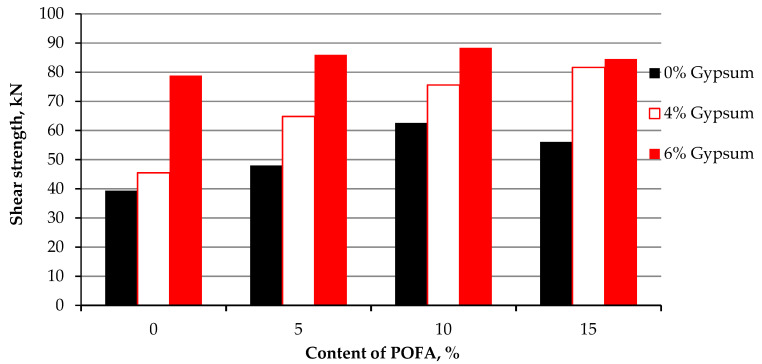
Shear strength of kaolin and stabilized kaolin with gypsum and POFA with no curing—on the 1st day.

**Figure 10:**
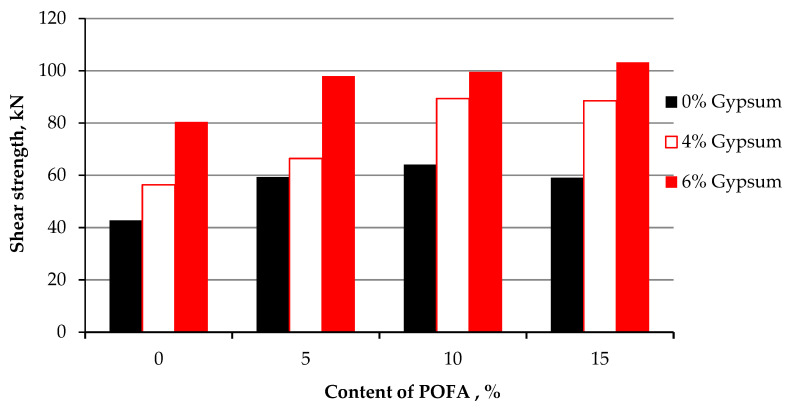
Shear strength of kaolin and treated kaolin with gypsum and POFA tested after 1 day of curing.

The results of treated kaolin are compared to the original kaolin. Figure 9 shows that the lowest increment of shear is achieved at KG4, which is only 6.41 kPa higher than the solo clay. On the other hand, the highest increment is achieved on kaolin stabilized at KG6P10 of 48.99 kPa on the first day of testing with no curing. The enhancement of shear strength is achieved because of the compaction effort, the different particle sizes of POFA, gypsum, and kaolin, as well as the chemical reaction of lime, which exists in high amounts in gypsum. The presence of the binary mixture in the treated soft clay within clay increases its shear strength. The increment of shear strength is attributed to the physicochemical and highly pozzolanic properties of the admixtures and the reduction of the plasticity index, which might make the mixture with clay content behave like granular soil (Figure 10), as mentioned by Onyelowe and Duc [91] by referring it to the reduction of water content and pozzolanic reactions. This encourages stabilization and the physical properties of POFA and gypsum, which are coarser than kaolin particles. Raising the shear strength has been gradually and consistently improved with curing time. The cohesion and interaction between the mixture were enhanced after compacting and increased with the time. Table 3 and Table 4 tabulate the variance and standard deviation of the specimen of shear strength on the 1st day and after the one day, respectively.

**Figure 11:**
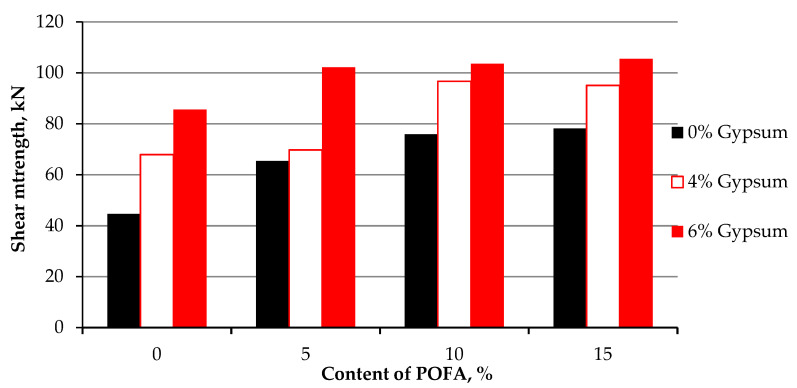
Stress–strain curve of kaolin and stabilized kaolin with gypsum and POFA tested after 7 days of curing.

All samples were tested at 7 curing days. The effect of gypsum and POFA on the strength of treated soil is illustrated in Figure 11. It was apparent that the shear strength was enhanced, and better results were shown than at first-day testing and 24 h of curing time. The enhancement of shear strength referred to the reaction between POFA, gypsum, and kaolin particles responsible for inter-particle bonding and bridging. Utilizing gypsum as a cementation agent may enhance clay particle aggregation, which led to an increase in shear strength and cohesion, which agreed with the results found by Rahman [92] on synthetic gypsum and fly ash for stabilizing clay soil. Table 5 summaries the variance and standard deviation of the specimen of shear strength after 7 days curing.

**Figure 12:**
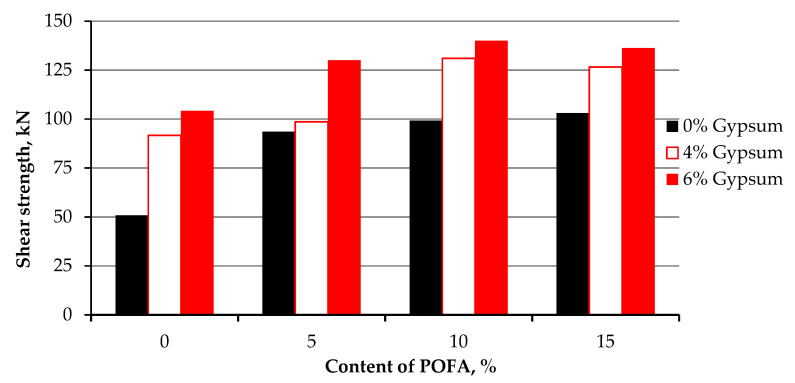
Stress–strain curve of kaolin and stabilized kaolin with gypsum and POFA tested after 28 days of curing.

In general, the outcome of this experimental work indicates that the pozzolanic reaction depends on the curing time (Figure 12). According to the findings, the particles bonding with POFA increased with the curing time. Since POFA contains a high amount of silica, the reaction time is delayed due to the low reactivity of silica and aluminium in POFA, which supports the statement by Teing et al. [45] Thus, a short curing time results in a low enhancement of shear strength in the treated kaolin. As gypsum contains a high amount of lime (CaO), the results illustrate that the strength is raised significantly, even with less curing time due to its high reactivity compared to silica and alumina.

It is reported that when POFA only is mixed with clay, reactions occur by two responsible mechanisms for strength improvement, modification (ion exchange), and stabilization (slow clay–POFA pozzolanic reactions) [38]. Table 6 summaries the variance and standard deviation of the specimen of shear strength after 28 curing days.

### 4.4. Shear Strength with Curing Period

**Figure 13:**
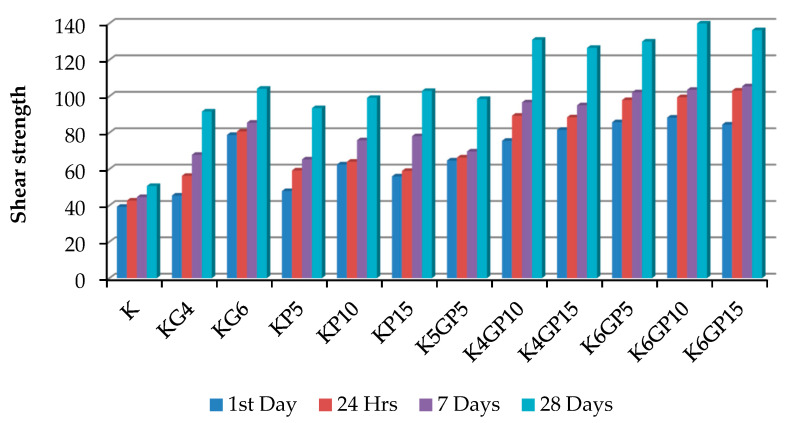
Stress-strain curve of kaolin and stabilized kaolin with gypsum and POFA at different curing times.

The improvement of shear strength in this study refers to many factors, mainly the pozzolanic reaction, which takes place with the curing of the sample before conducting the testing, and the amount of stabilizing agents of POFA and gypsum (Figure 13). Overall, the enhancement of shear strength with the curing time is clear as the pozzolanic reaction takes place with the curing time.

### 4.5. Microstructural Imaging

**Figure 14:**
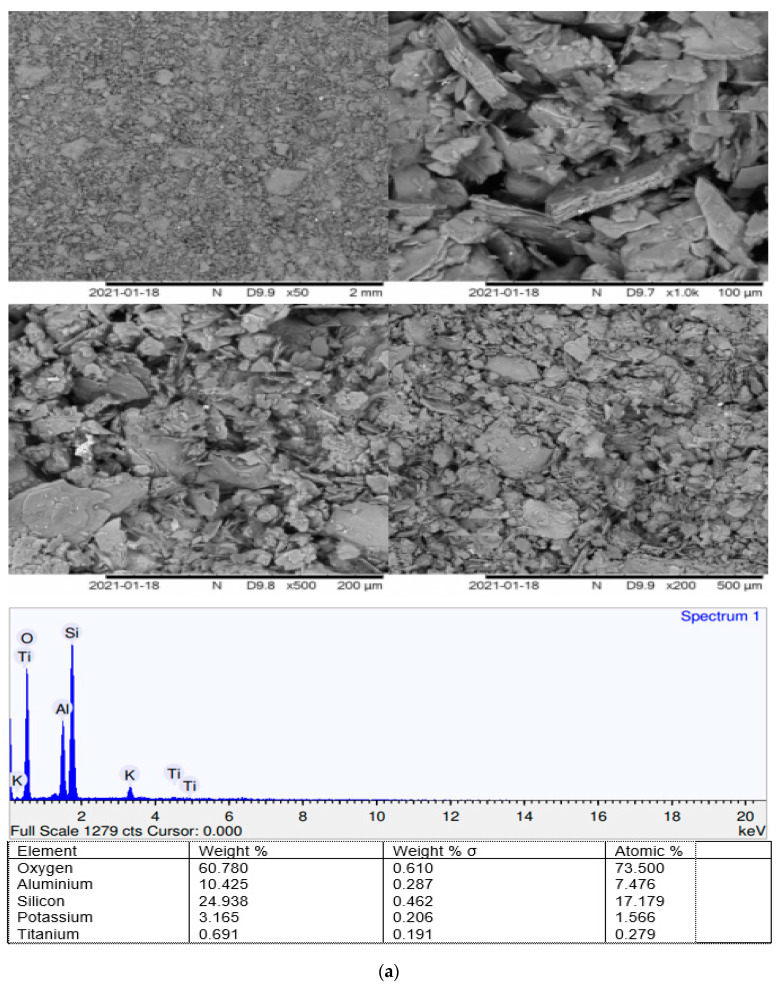

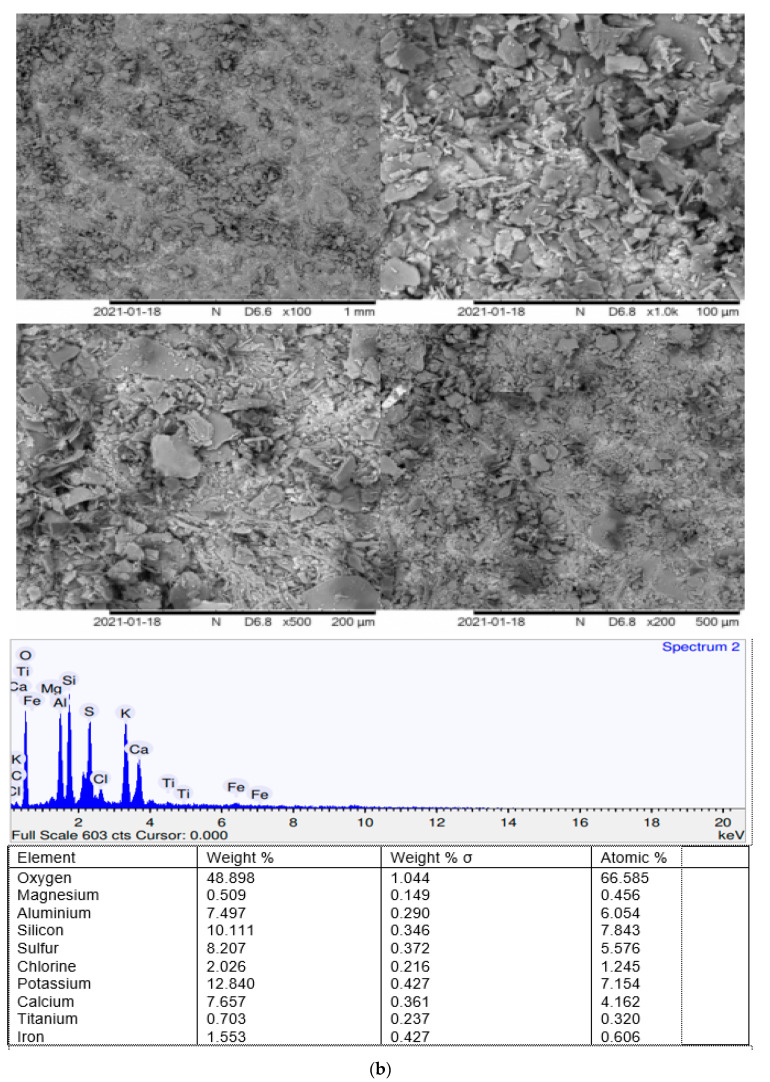
(**a**) SEM and EDX of kaolin and treated kaolin 28 days of curing. (**b**) SEM and EDX of treated kaolin of KG6P10 at 28 days of curing.

The results from SEM for the control sample are shown in the Figure 14a,b. The image of the microstructure shows clear voids on untreated kaolin. EDX shows compatible results with the results obtained from SEM. Oxygen shows around 60.78% of the elemental weight and 73.5% of the atomic weight, which verifies the high number of voids in the compacted kaolin. The SEM results also show no product of hydration on untreated kaolin. The results from EDX in the Figures also show that there is no calcium. Aluminium is also very low where both are the key elements to produce hydration products such as Ettringite, Portlandite (CH), and cementitious gel (C-S-H). Similar results are found by Jawad [93] for utilizing POFA with calcium carbide. The photomicrographs of the control sample, the flaky shaped particles which represent the clay and other minerals in the soil are easily recognized and described [94]. Overall, no hydration has occurred for the control sample where voids are easily recognized and distinguished.

The results from SEM for the gypsum–POFA-treated sample are shown in the Figures. A change has occurred where the images of microstructure show a clear reduction of voids on kaolin treated with 6% gypsum and 10% POFA (KG6P10). The SEM results also illustrate a clear hydration process of stabilization, where the existing CSH (flake-shape particles) and CAH (needle-shaped crystals) gels as poof of gypsum assist in forming ettringite crystals [95], resulting in enhanced soil compressive and shear strengths.

EDX also shows compatible results with those obtained from SEM. The results from EDX show that the oxygen weight reduces for cross-section and surface scanning to about 58.02% and 48.898% of the elemental weight and 72.01% and 66.585, sequentially. Calcium is found to weigh 3.23% and 1.6% of the atomic weight for cross-section scanning as well as the weight of 7.657% and 4.162% of atomic weight for surface scanning, where the control sample does not exist. Iron is also found with the weight of 1.56% and 1.553% and 0.44% and 0.606% of the atomic weight for cross-section and surface scanning, sequentially, which verifies that the key elements of cementitious gel (C-S-H) and Ettringite Portlandite (CH) exist in the treated kaolin. The calcium peak in the graph indicates the existence of CSH and CAH gels in the treated sample [38,96]. Similar results are found by Jawad [93] for utilizing POFA with calcium carbide. POFA-gypsum mixture appears to have some irregular and spherical-shaped particles with sharp angles, fewer voids, and apparent enhancement of hydration products.

## 5. Conclusions

Compaction proctor test (SPT) is an important geotechnical in this research as an unconfined compression test (UCT) depends on the results obtained from this test. The result of the SPT test exhibited an overall increase in OMC by substituting more POFAs and decrements of MDD, which was interpreted due to the low POFA specific gravity; meanwhile, findings did not show a noteworthy water content increment when kaolin was treated with gypsum only.

Unconfined compression test results show a clear enhancement of soil compressive strength. The strength increases and is affected by both curing days and POFA–gypsum mixture and particle size of both stabilizer and kaolin. Kaolin alone does not affect the curing time much. This proves that the pozzolanic reaction enhances the strength because of the formation of various calcium silicate hydrates and calcium aluminate hydrates, which depend on the curing and reaction condition, water content, and mineralogy of clay and stabilizing agents. Shear strength measurements were controlled and dependent on various factors, such as sample handling, mixing, pozzolanic reaction, and the amount of stabilizing agents. The results show a clear relationship of soil shear strength improvement with both stabilizing agent dosage and curing time with a positive, strong, and high coefficient of determination.

The clear change on scanning electron microscope images of untreated and treated kaolin, the weight percent of chemical compositions obtained from the X-ray fluorescence test of original study materials, and the weight and presence of chemical elements of original and stabilized kaolin by Energy-dispersive X-ray spectroscopy test shows compatible results to verify and justify the reasons behind shear strength improvement such as (1) reduction of the voids of KG6P10-treated kaolin compared to the control kaolin sample; (2) the existance of the hydration product of the treated sample; and (3) the change in the size and shape of particles after treatment. However, based on the findings of this study, several concluding points have been drawn as follows:

- The reduction of MDD during soil stabilization is about 10%, which is referred to due to the compaction resistance caused by the flocculation of mixture particles.
- In the case when POFA is used as a stabilizing agent with gypsum, it is found that the density of soft clay is affected by lime content, existing in gypsum, where the lime has the tendency to absorb more water and hence decrease the MDD of treated soil.
- The increment of the strength with the mixture of gypsum and POFA is also predicted to double compared to the control sample due to soil susceptibility to water content variation reduction. This is followed by agglomeration and flocculation of soil particles.
- Utilizing gypsum as a cementation agent may enhance clay particle aggregation, which led to an increase in shear strength and cohesion on synthetic gypsum and POFA for stabilizing clay.
- As gypsum contains a high amount of lime (CaO), the results illustrate that the strength is raised significantly, even with less curing time due to its high reactivity compared to silica and alumina.
- POFA–gypsum mixture appears to have some irregular and spherical-shaped particles with sharp angles, fewer voids, and an apparent enhancement of hydration products.
- Overall, no hydration has occurred for the control sample where voids are easily recognized and distinguished, where the treated sample clearly showed hydration products.

Based on the major findings of this study, further studies are suggested to treat POFA under different conditions to stabilize soil. Utilizing nano–POFA by grinding it, by using a grinding machine such as the Los Angeles abrasion machine (LAAM), for many cycles yields a fine particle size, or by utilizing an electric furnace to remove the unburned carbon. In this study, The gypsum was used as 4% and 6% to be mixed with POFA. This study could be continued to determine the optimum amount of gypsum based on the improvement of shear strength under the UCT test. Applying the optimum amount with POFA waste helps to achieve better results. It is also recommended to conduct consolidated undrained triaxial tests for these reported materails to further study other parameters such as pore water pressure, the angle of friction and more, which can be defined through the triaxial test as compared to the UCT test.

## Figures and Tables

**Table 1:** Chemical Properties of POFA, gypsum and kaolin.

Material/Chemical Compound Name	Chemical Formula	POFA	Gypsum	Kaolin
Alumina	Al_2_O_3_	1.33	1.25	17.1
Ferrite	Fe_2_O_3_	8.71	0.422	0.626
Silica	SiO_2_	35.9	4.97	73.5
Lime	CaO	13.2	47	-
Potassium Oxide	K_2_O	35.4	0.756	7.23
Magnesia	MgO	1.24	0.816	0.79
Sulfur trioxide	SO_3_	1.39	44.6	0.102
Titanium dioxide	TiO_2_	-	-	0.343
Phosphorus Pentoxide	P_2_O_5_	1.91	0.164	-
Manganese (II) oxide	MnO	0.257	-	-
Chlorine	Cl	0.256	-	-

**Table 2:** Precentages of the utilized stabilizing agents with mass, density and volume.

Code	POFA	Gypsum	Kaolin	OMC %	Compacted Sample Mass (g)	ρ	Volume
K	0	0	100	18	165.69	1.922	
KG4	0	4	96	18.3	165.2075	1.917	
KG6	0	6	94	18.8	166.4075	1.931	
KP5	5	0	95	23	162.555	1.886	
KP10	10	0	90	24	159.185	1.847	
KP15	15	0	85	19.2	151.44	1.757	86.19
KG4P5	5	4	91	19.8	159.5875	1.852	
KG4P10	10	4	86	19.3	157.185	1.824	
KG4P15	15	4	81	19.20	154.5225	1.793	
KG6P5	5	6	89	19.10	161.235	1.871	
KG6P10	10	6	84	19.20	157.58	1.828	
KG6P15	15	6	79	19.00	157.625	1.829	

**Table 3:** Summary of variance and standard deviation of the specimen of shear strength on the 1st day.

Sample	Variance	Standard Deviation	Sample	Variance	Standard Deviation
K	0.017	0.67	K4GP5	0.012	0.46
KG4	0.009	0.37	K4GP10	0.032	0.78
KG6	0.014	0.060	K4GP15	0.006	0.25
KP5	0.022	0.87	K6GP5	0.0032	0.124
KP10	0.0063	0.25	K6GP10	0.012	0.44
KP15	0.0057	0.23	K6GP15	0.002	0.033

**Table 4:** Summary of variance and standard deviation of the specimen of shear strength after one day curing.

Sample	Variance	Standard Deviation	Sample	Variance	Standard Deviation
K	0.025	0.82	K4GP5	0.06	0.73
KG4	0.0025	0.062	K4GP10	0.09	1.55
KG6	0.023	0.63	K4GP15	0.009	0.19
KP5	0.051	1.22	K6GP5	0.04	0.46
KP10	0.042	1.18	K6GP10	0.007	0.28
KP15	0.028	1.22	K6GP15	0.005	0.49

**Table 5:** Summary of variance and standard deviation of the specimen of shear strength after 7 curing days.

Sample	Variance	Standard Deviation	Sample	Variance	Standard Deviation
K	0.038	0.68	K4GP5	0.012	0.46
KG4	0.071	0.71	K4GP10	0.032	0.78
KG6	0.0032	0.072	K4GP15	0.006	0.25
KP5	0.021	0.78	K6GP5	0.0032	0.124
KP10	0.017	0.23	K6GP10	0.012	0.44
KP15	0.017	0.28	K6GP15	0.002	0.033

**Table 6:** Summary of variance and standard deviation of the specimen of shear strength after 28 curing days.

Sample	Variance	Standard Deviation	Sample	Variance	Standard Deviation
K	0.027	0.79	K4GP5	0.009	0.67
KG4	0.019	0.78	K4GP10	0.024	0.78
KG6	0.008	0.34	K4GP15	0.017	0.099
KP5	0.024	0.26	K6GP5	0.0025	0.16
KP10	0.003	0.89	K6GP10	0.028	0.32
KP15	0.006	0.44	K6GP15	0.009	0.053

## Data Availability

Data sharing not applicable.
